# Supplementary material for: Assessing intra- and inter-molecular charge transfer excitations in non-fullerene acceptors using electroabsorption spectroscopy
Source: Nat Commun. 2024 Mar 16;15:2393. doi: 10.1038/s41467-024-46462-x (PMC10944474; doi:10.1038/s41467-024-46462-x)
Supplement: Supplementary file 1 — Supplementary Information [file 41467_2024_46462_MOESM1_ESM.pdf]

## Supplementary Information

# Assessing Intra- and Inter-Molecular Charge Transfer Excitations in Non-Fullerene Acceptors Using Electroabsorption Spectroscopy

*Sudhi Mahadevan[1,2,3], Taili Liu[4], Saied Md Pratik[5], Yuhao Li[6], Hang Yuen Ho[1,2,3], Shanchao Ouyang[1,2,3], Xinhui Lu[6], Hin-Lap Yip[1,2,3,7], Philip C.Y Chow[8], Jean-Luc Brédas[5], Veaceslav Coropceanu[5], Shu Kong So[9], Sai-Wing Tsang[1,2,3,*]*

**AFFILIATIONS**

[1] Department of Materials Science and Engineering, City University of Hong Kong, Hong Kong SAR, P.R. China.

[2] Centre of Super-Diamond and Advanced Films, City University of Hong Kong, Hong Kong SAR, P.R. China.

[3] Hong Kong Institute of Clean Energy, City University of Hong Kong, Hong Kong SAR, P.R. China.

[4] College of Physics and Electronic Information, Yunnan Normal University, Yunnan Kunming 650500, China.

[5] Department of Chemistry and Biochemistry, The University of Arizona, Tucson, Arizona 85721-0041, United States.

[6] Department of Physics, The Chinese University of Hong Kong, Hong Kong SAR, P. R. China.

[7] School of Energy and Environment, City University of Hong Kong, Hong Kong SAR, P.R. China.

[8] Department of Mechanical Engineering, The University of Hong Kong, Pok Fu Lam, Hong Kong SAR, P. R. China.

[9] Department of Physics and Institute of Advanced Materials, Hong Kong Baptist University, Kowloon Tong, Hong Kong SAR, P. R. China.

*Author to whom correspondence should be addressed: saitsang@cityu.edu.hk

# Outline


| Supplementary Figures and Tables | Page No. |
|---|---|
| **Supplementary Fig. 1:** Stark effect and change in optical absorption | 5 |
| **Supplementary Fig. 2:** Solid-solvation method to isolate Y6 in thin films using PVK, PS, and PMMA | 7 |
| **Supplementary Fig. 3:** Illustration of two distinct dimer configurations of ITIC, labeled as A and B, extracted from the crystal structure | 8 |
| **Supplementary Fig. 4:** DFT results for ITIC | |
| **a.** Natural Transition Orbitals (NTOs) representing the hole and electron distributions in the lowest singlet excited state of dimer A of ITIC | 8 |
| **b.** Natural Transition Orbitals (NTOs) representing the hole and electron distributions in the lowest singlet excited state of dimer B of ITIC | 9 |
| **Supplementary Fig. 5:** GIWAS & GISAXS results of different loading ratios of Y6 in PVK | 10 |
| **Supplementary Fig. 6:** Optical absorption fitting using Frank - Condon principle | 13 |
| **Supplementary Fig. 7:** DFT results for Y6 | 16 |
| **Supplementary Fig. 8:** Illustration of two distinct dimer configurations of Y6 | 16 |
| **a.** Natural Transition Orbitals (NTOs) representing the hole and electron distributions in the lowest four singlet excited states of dimer A of Y6 | 16 |
| **b.** Natural Transition Orbitals (NTOs) representing the hole and electron distributions in the lowest four singlet excited states of dimer B of Y6 | 17 |
| **c.** Natural Transition Orbitals (NTOs) representing the hole and electron distributions in the lowest four singlet excited states of dimer C of Y6 | 17 |
| **Supplementary Fig. 9:** Part by Part EA fitting at different aggregate and non-interacting molecular regions based on Franck- Condon progressions | 21 |
| **a.** Electroabsorption spectrum fitting of 10 wt% Y6 | 21 |
| **b.** Electroabsorption spectrum fitting of 100 wt% Y6 | 22 |

**Supplementary Fig. 10: a.** Energy diagram showing Stark effect

**b.** Change in absorbance with and without the influence of external electric field 23

**Supplementary Fig. 11: First and second harmonic DC bias dependence EA results**

**a.** First harmonic DC bias dependence EA Spectra of dispersed and pristine Y6 and ITIC thin films 29

**b.** First harmonic DC bias dependence of EA signals at different loading ratios of Y6 in the PVK polymer matrix 30

**c.** Second harmonic DC bias dependence of EA signals at different loading ratios of Y6 and ITIC in PVK polymer matrix 31

**d.** First harmonic DC bias dependence of EA signals of pristine Y6 with different values of anisotropic or static dipole moment 32

**Supplementary Fig. 12:** Thin film absorbance fitting using Gaussian deconvolution 35

**Supplementary Table 1.** Morphology parameters fitted by GISAXS profiles 12

**Supplementary Table 2.** The parameters for Frank-Condon progressions for different loading ratios of Y6 15

**Supplementary Table 3.a.** Calculated energies for the lowest four singlet excited states ($S_1$ to $S_4$, denoted as ΔE) of dimer A of Y6 18

**Supplementary Table 3.b.** Calculated energies for the lowest four singlet excited states ($S_1$ to $S_4$, denoted as ΔE) of dimer B of Y6 18

**Supplementary Table 3.c.** Calculated energies for the lowest four singlet excited states ($S_1$ to $S_4$, denoted as ΔE) of dimer C of Y6 19

**Supplementary Table 4.a.** Calculated energies for the lowest four singlet excited states ($S_1$ to $S_4$, denoted as ΔE) of dimer A of ITIC 19

**Supplementary Table 4.b.** Calculated energies for the lowest four singlet excited states ($S_1$ to $S_4$, denoted as ΔE) of dimer B of ITIC 20

**Supplementary Table 5.** Calculation of linear dipoles from first harmonic DC bias 33

dependence of EA signals at different loading ratios of Y6 in PVK polymer matrix
**Supplementary Note 1:** Mathematical derivation of $EA_{1\omega}$ and $EA_{2\omega}$ fitting equations 23
**Supplementary Note 2:** First and second harmonic DC bias dependence EA results 28
**Supplementary Note 3:** Fitting procedure of $EA_{2\omega}$ spectrum using Gaussian deconvolution method 34
**Supplementary Note 4:** Four-State Model 36
**Supplementary References** 37

## Supplementary Figures and Tables

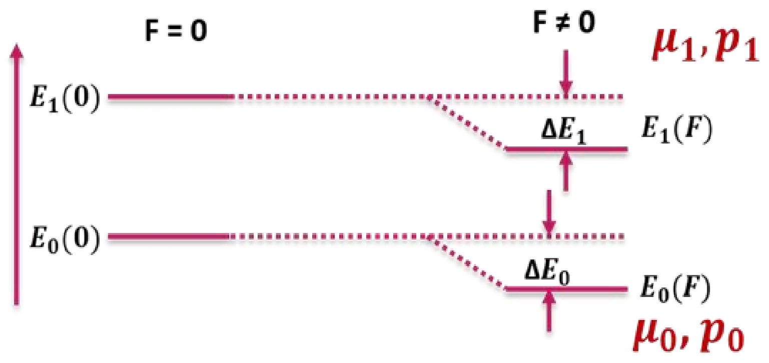


- *Stark effect and change in optical absorption.*

**Supplementary Fig. 1:** Energy diagram showing Stark effect.

Energy diagram without and with electric field. $E_0(0)$ and $E_0$(F) represent ground-state energies with and without electric field, respectively. $E_1$ (0) and $E_1$ (F) represent excited-state energies with and without electric field, respectively. $\Delta E_1$ and $\Delta E_0$ stand for excited-state and ground-state energy shifts, respectively. The Y-axis on the left defines the increasing direction of energy (eV).

According to the Stark effect, the energy level of a state will shift under an electric field (F) by a factor related to the dipole moment (μ) and polarizability (p) in its equilibrium state[1]:

$$E(F) \approx E\,(0) - \mu.F - \frac{1}{2}\,p.F^2 \quad (1)$$

Equation (1) can be written for the excited and ground states as:

Excited state $$E_1(F) = E_1\,(0) - \mu_1\,F - \frac{1}{2}\,p_1.F^2 \quad (2)$$

Ground state $$E_0\,(F) = E_0\,(0) - \mu_0\,F - \frac{1}{2}\,p_0.F^2 \quad (3)$$

Equations (2) – (3) combine to give the relative energy shift between the excited and ground states:

$$\Delta E = [E_0(F) - E_0(0)] - [E_1(F) - E_1(0)]$$

$$= -[\mu_1 - \mu_0]F - \frac{1}{2}F^2[p_{1-}p_0] \quad (4)$$

$$\Delta E = -\Delta\mu F - \Delta p\frac{F^2}{2} \quad (5)$$

ΔE indicates the relative shift (shifting parameter) in excitation energy upon application of the electric field. Δμ= $(\mu_1 - \mu_0)$, the difference in permanent dipole moment (unit - Debye) between ground and excited states. Δp = $(p_1 - p_0)$, the difference in polarizability (unit – $cm^3$) between ground and excited states. Consequently, measuring the change in absorption under an electrical field (electroabsorption, EA) brings insight into the excitonic properties (Δμ and Δp) of a material. A detailed mathematical derivation of the first and second-harmonic EA equations is given in Supplementary Note 1.

- *Solid-solvation method to isolate Y6 in thin films using PVK, PS, and PMMA*

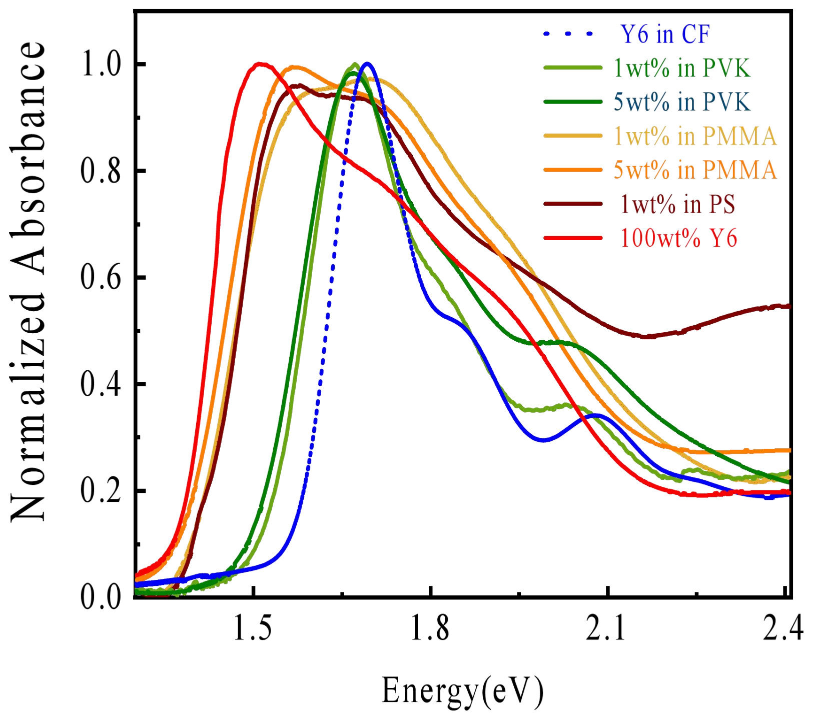


**Supplementary Fig. 2:** UV-vis absorption spectra of thin films having Y6 dispersed in PS, PMMA, and PVK.

- *DFT results for ITIC.*

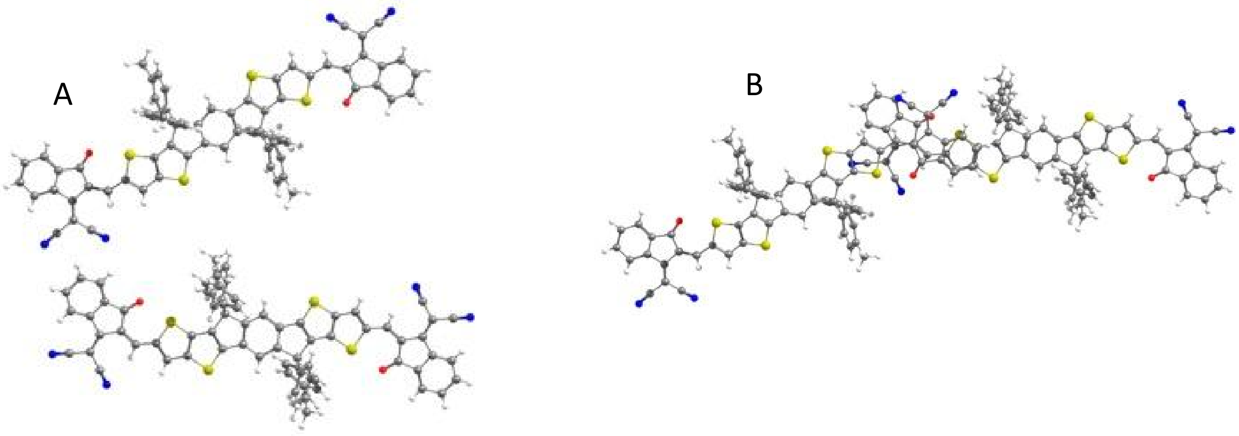


**Supplementary Fig. 3:** Illustration of two distinct dimer configurations of ITIC, labeled as A and B, extracted from the crystal structure reported in the literature[2]. Both dimers have symmetric constituent monomers. We replaced the long side chains with -$CH_3$ groups for our calculations.

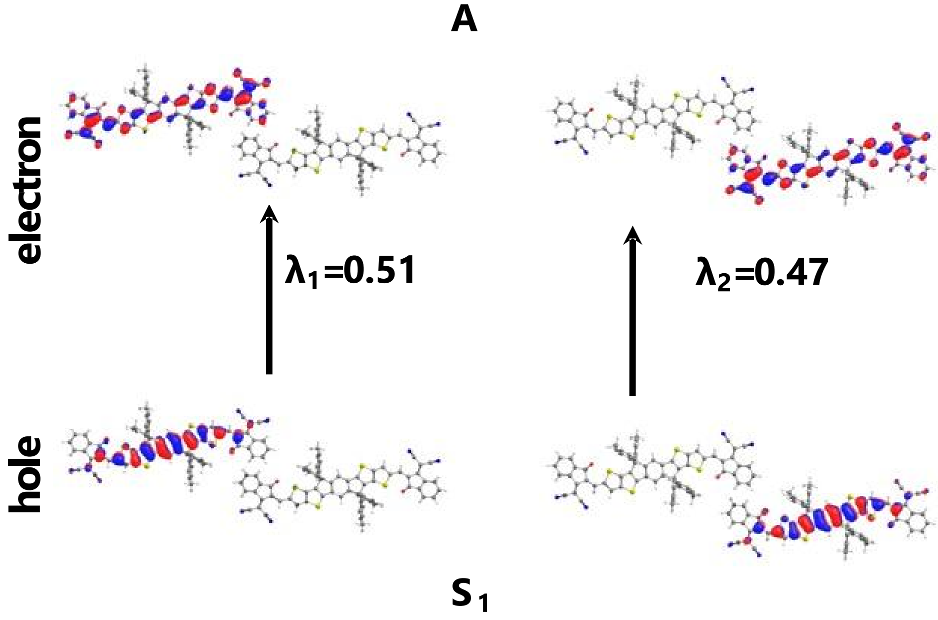


**Supplementary Fig. 4.a:** Natural Transition Orbitals (NTOs) representing the hole and electron distributions in the lowest singlet excited state of dimer A of ITIC.

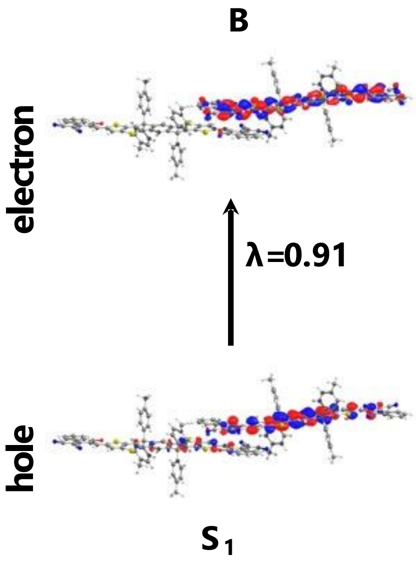


**Supplementary Fig. 4.b:** Natural Transition Orbitals (NTOs) representing the hole and electron distributions in the lowest singlet excited states of dimer B of ITIC.

- *GIWAS & GISAXS results of different loading ratios of Y6 in PVK*

To identify the stacking change of PVK and Y6, we use *, #, and + symbols to represent the signals from PVK, Y6, and unclarified, respectively. First, the peaks of PVK (*) shift to the lower q region with an increase in Y6 loading, indicating the stacking between PVK chains is getting looser. It indicates the stronger aggregation of Y6 in the thin film. Second, the π-π peak of Y6 (*) shifts to a higher 'q' region from 70% Y6 loading to pure film, indicating the stacking between the Y6 is getting tighter. Moreover, the peaks labeled by '+' show systematic shifting to the lower q region with the increase in Y6 loading; however, due to the low resolution and overlapping of different peaks, it is challenging to judge whether this peak belongs to PVK or Y6.

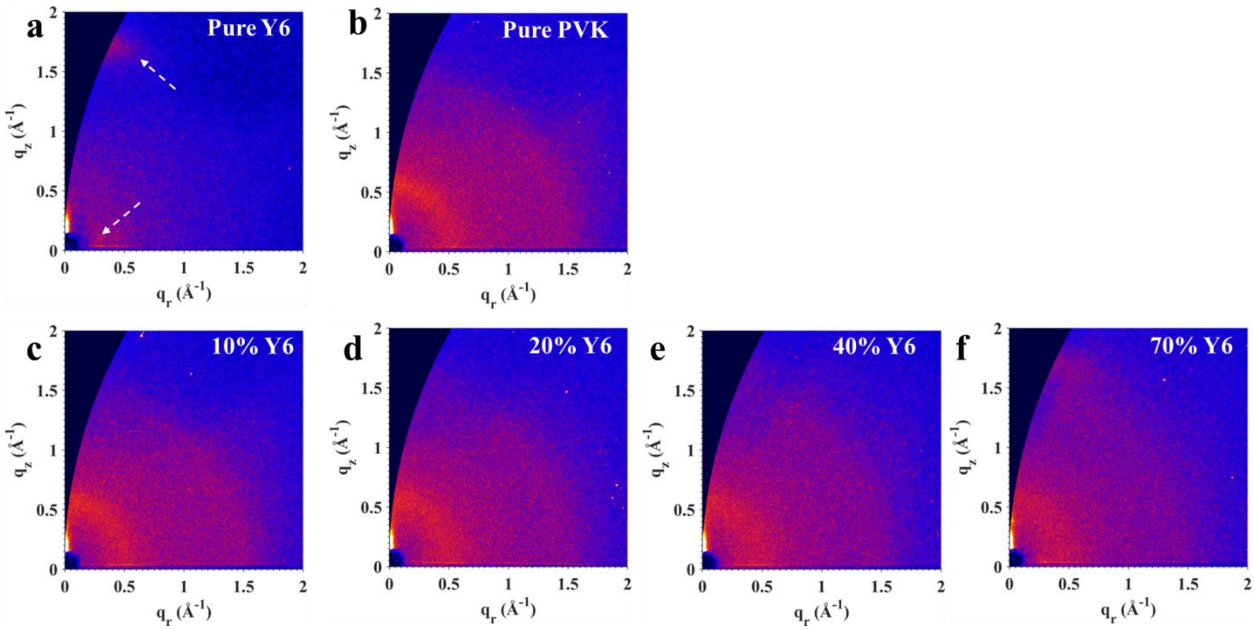

g

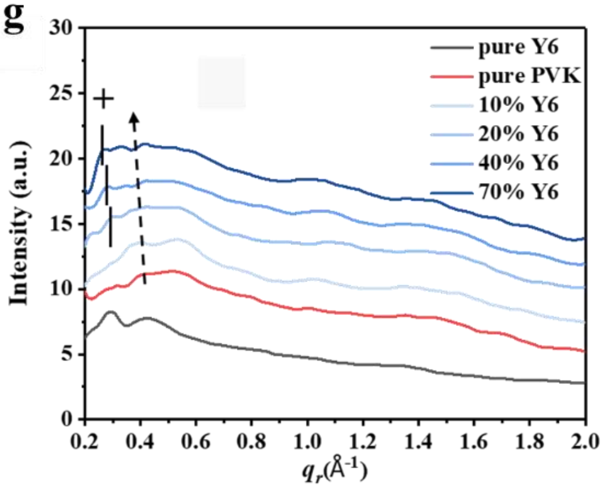
pure Y6
pure PVK
10% Y6
20% Y6
40% Y6
70% Y6
+
Intensity (a.u.)
$q_r$(Å$^{-1}$)

h

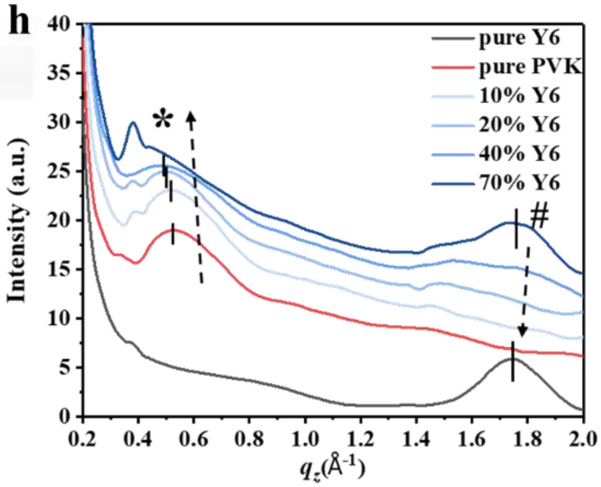
pure Y6
pure PVK
10% Y6
20% Y6
40% Y6
70% Y6
*
#
Intensity (a.u.)
$q_z$(Å$^{-1}$)

i

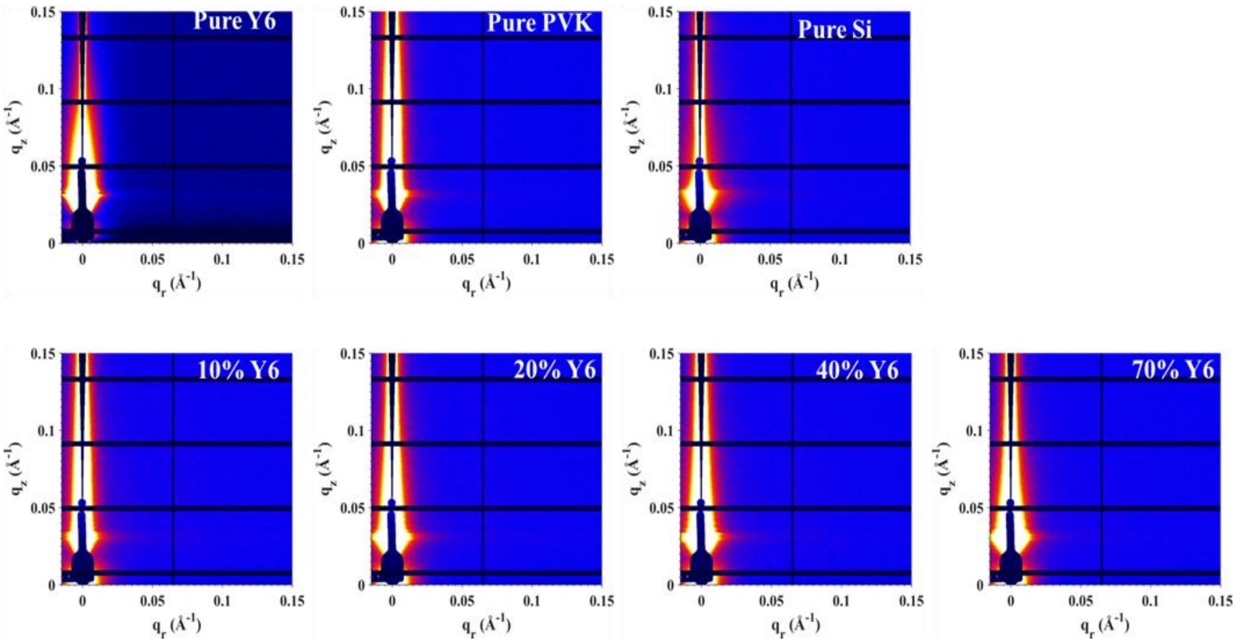
Pure Y6
Pure PVK
Pure Si
10% Y6
20% Y6
40% Y6
70% Y6
$q_z$ (Å$^{-1}$)
$q_r$ (Å$^{-1}$)

j

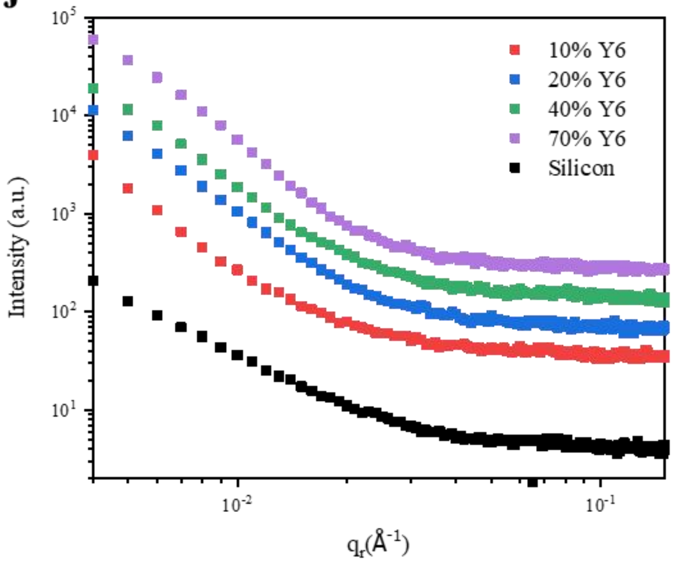


**Supplementary Fig. 5: a-f** 2D GIWAXS patterns of Y6 films and **g, h** corresponding GIWAXS intensity profiles along the in-plane and out-of-plane directions, respectively. The symbols *, #, and + represent the signals from PVK, Y6, and unclarified, respectively. Graphs **i and j** show the 2D GISAXS patterns from the synchrotron test and GISAXS intensity profiles along the in-plane (IP) direction, respectively.

**Supplementary Table 1:** Morphology parameters fitted by GISAXS profiles (ξ is the domain size of the amorphous phase).

| Chemicals | ξ (nm) |
|---|---|
| Pure PVK | 37.4 |
| 10% Y6 | 33.6 |
| 20% Y6 | 21.5 |
| 40% Y6 | 22.2 |
| 70% Y6 | 21.6 |

- *Optical absorption fitting using Frank Condon principle.*

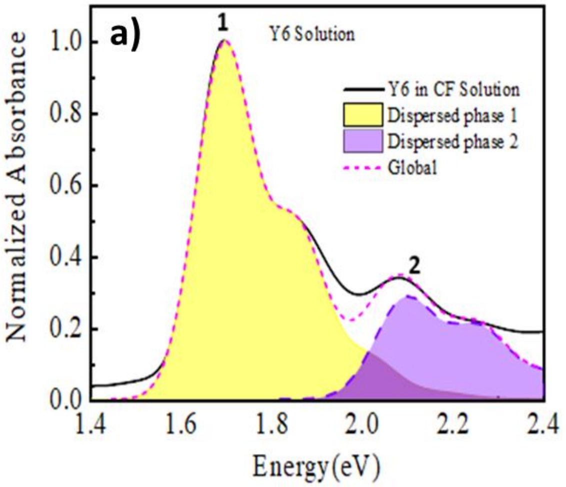


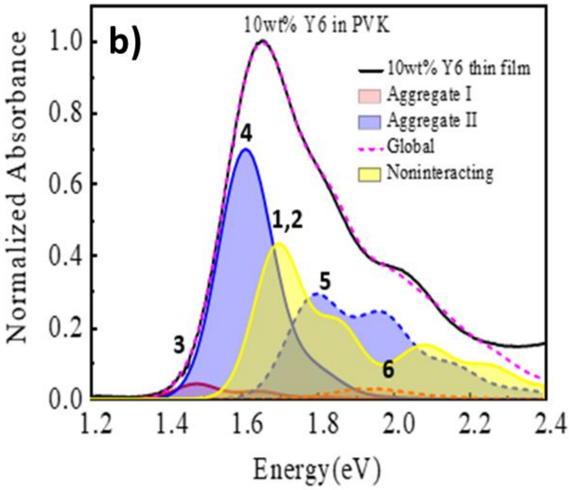


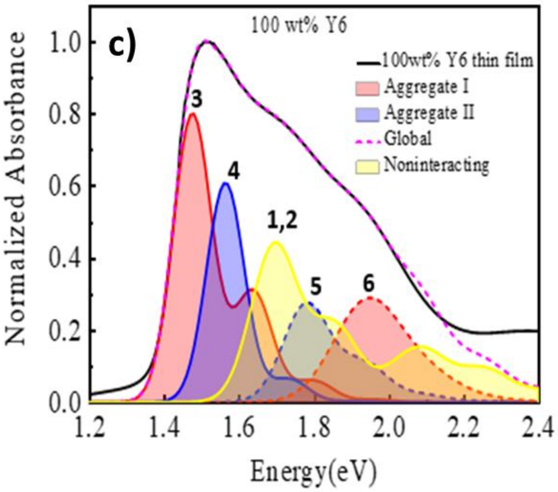


**Supplementary Fig. 6:** Fitted absorption spectra of Y6 in **a)** CF solution (20-25 mg/ml), **b)** 10wt% Y6 in PVK, and **c)** 100wt% Y6 thin films with the Frank-Condon-weight density of states (FCWD) in the framework of Marcus-Levich-Jortner theory (detailed description in text). Dispersed phases containing non-interacting molecules are marked as 1 and 2 (colored in yellow). ‘Aggregate I’ is marked as 3 and 6 (colored in red), and ‘Aggregate II’ is marked as 4 and 5 (colored in blue), respectively. Both have progressions in the low-energy (solid line) and high-energy (dashed line) regions. The global fit is indicated as a pink dashed line.

According to Kasha’s model, the interaction between adjacent molecules leads to the energetic splitting of the excited states of the monomer[3]. As shown in the figure, we first fitted the optical

absorption of the Y6 solution, where the spectrum predominantly contained non-interacting molecules (dispersed phases 1 and 2). The fitting equation of the absorption spectra with the Frank-Condon-weight density of states (FCWD) in the framework of Marcus-Levich-Jortner theory[4] is expressed as:

$$FCWD = \frac{1}{\sqrt{4\pi\lambda k_B}} \sum_{n=0}^{\infty} exp(-S) \frac{S^n}{n!} exp\left[-\frac{(\Delta E + n\hbar\omega + \lambda)^2}{4\lambda k_B}\right] \tag{6}$$

where $\lambda$ is the Marcus reorganization energy (meV), S is the Huang-Rhys factor accounting for the coupling of the two states, $k_B$ is Boltzmann constant, and $\Delta E$ is the energy difference between the oscillation energy and 0-0 transition energy $E_{00}$ (eV). Two FC progressions with transition energies determined by the peak local maxima at around 1.69 eV ($E_{01}$) and 2.1 eV ($E_{02}$) can be well reproduced. A vibrational energy, $h\omega_1$ = 160 meV, was used on the Raman measurements of Y6[5]. The peak intensity values (A), Huang-Rhys parameter (S), and Gaussian linewidth parameter (λ) were adjusted within a reasonable range of values for different transitions to obtain a good fit.

For thin films having 10 wt% and 100 wt% Y6, we kept the FC parameters of the Y6 solution constant (peaks 1 and 2); four more FC progressions at transition energies ( $E_{03}$- $E_{06}$) can be found. Since each progression represents the electronic configuration of isolated molecules and different dimers, the FC parameter should be similar in different samples. During the fitting, the values of the transition energies (E), the vibrational energy (hω), the Huang-Rhys parameter ($S_1$), and the Gaussian linewidth parameter (λ) for each progression were kept almost the same for all samples. Only the peak intensity values (A3 to A6) were adjusted to get the best spectral fit. These fitting results and FC parameters we obtained are very consistent with the results obtained by Köhler and co-workers[6]. The table below shows the FC parameters used for different loading ratios of Y6.

**Supplementary Table 2**: Parameters used for the FC progressions at different loading ratios of Y6. ($k_B$T = 25.9 meV)

| No. | Parameters | Y6 Solution | 10 wt% Y6 thin film | 100 wt% Y6 thin film |
|---|---|---|---|---|
| 1 | A1 | 248 | 248 | 248 |
| | $\lambda_1$ (meV) | 75 | 75 | 75 |
| | S1 | 0.48 | 0.48 | 0.48 |
| | $E_{01}$ (eV) | 1.62 | 1.62 | 1.62 |
| | $h\omega_1$ (meV) | 160 | 160 | 160 |
| 2 | A2 | 93 | 93 | 93 |
| | $\lambda_2$ (meV) | 85 | 85 | 85 |
| | S2 | 0.71 | 0.71 | 0.71 |
| | $E_{02}$ (eV) | 2.01 | 2.01 | 2.01 |
| | $h\omega_2$ (meV) | 163 | 163 | 163 |
| 3 | A3 | - | 9.68 | 151 |
| | $\lambda_3$ (meV) | - | 60 | 51 |
| | S3 | - | 0.51 | 0.38 |
| | $E_{03}$ (eV) | - | 1.42 | 1.42 |
| | hω3 (meV) | - | 164 | 162 |
| 4 | A4 | - | 138 | 85.5 |
| | $\lambda_4$ (meV) | - | 96 | 49 |
| | S4 | - | 0.11 | 0.10 |
| | $E_{04}$ (eV) | - | 1.51 | 1.51 |
| | $h\omega_4$ (meV) | - | 180 | 170 |
| 5 | A5 | - | 114 | 62.3 |
| | $\lambda_5$ (meV) | - | 99.7 | 74.7 |
| | S5 | - | 0.81 | 0.38 |
| | $E_{05}$ (eV) | - | 1.69 | 1.71 |
| | $h\omega_5$ (meV) | - | 180 | 155 |
| 6 | A6 | - | 7.79 | 78.4 |
| | $\lambda_6$ (meV) | - | 150 | 150 |
| | S6 | - | 0.20 | 0.26 |
| | $E_{06}$ (eV) | - | 1.78 | 1.78 |
| | $h\omega_6$ (meV) | - | 170 | 155 |
| | A12 | - | 0.43 | 0.44 |

- <u>DFT results for Y6</u>

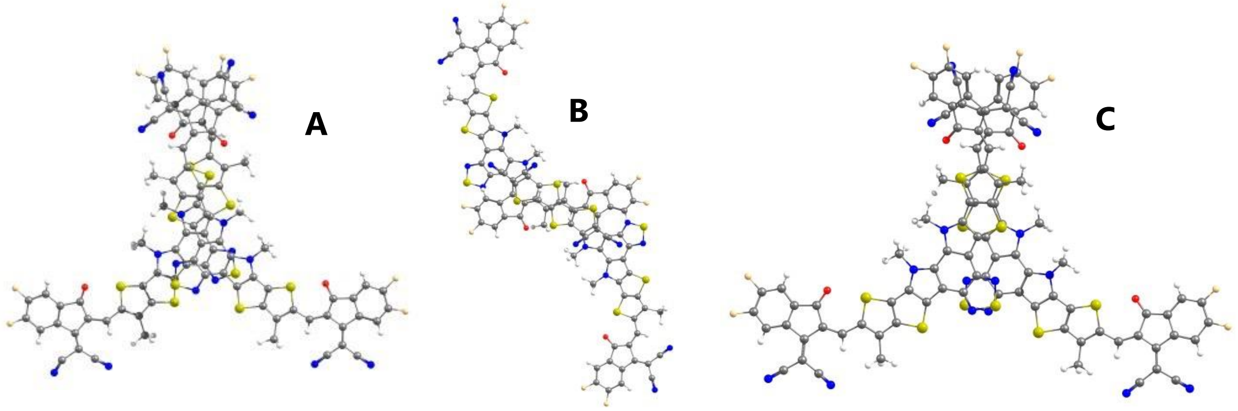


**Supplementary Fig. 7:** Illustration of two distinct dimer configurations of Y6, labeled as A and B, extracted from the crystal structure reported from our previous work[7] and Dimer C (which closely resembles Dimer A) retrieved from the work by Marks and co-workers[8]. Both dimers A and C comprise asymmetric constituent monomers, whereas dimer B features symmetric constituent monomers. We replaced the long side chains with -$CH_3$ groups for our calculations.

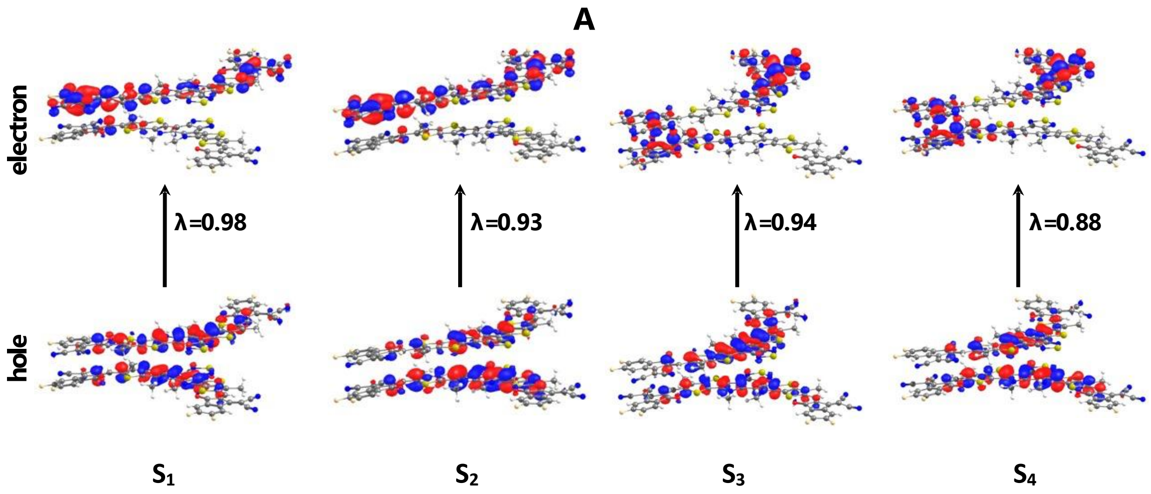


**Supplementary Fig. 8.a:** Natural Transition Orbitals (NTOs) representing the hole and electron distributions in the lowest four singlet excited states of dimer A of Y6.

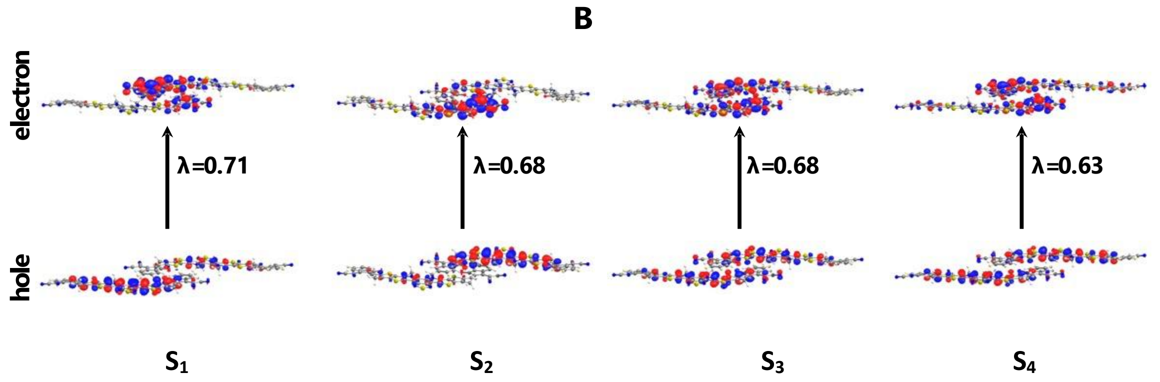


**Supplementary Fig. 8.b:** Natural Transition Orbitals (NTOs) representing the hole and electron distributions in the lowest four singlet excited states of dimer B of Y6.

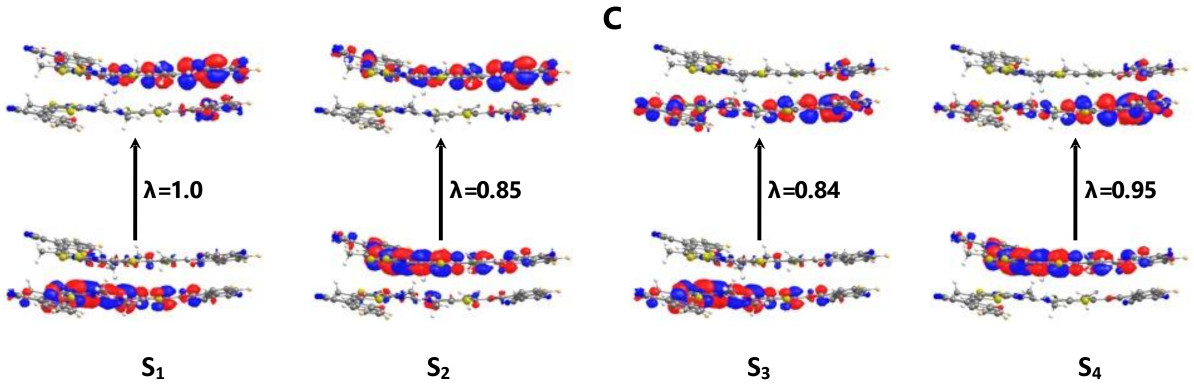


**Supplementary Fig. 8.c:** Natural Transition Orbitals (NTOs) representing the hole and electron distributions in the lowest four singlet excited states of dimer C of Y6.

**Supplementary Table 3.a:** Calculated energies for the lowest four singlet excited states ($S_1$ to $S_4$, denoted as ΔE) of dimer A of Y6, along with the corresponding oscillator strengths (f) for $S_0 \rightarrow S_n$ (n=1 to 4) transitions. The table also includes the variations in dipole moment (Δμ) and polarization (Δp) during these transitions. Additionally, similar values for the $S_1$ state are provided for the constituent monomeric units ($M_1$ and $M_2$), comprising dimer A of Y6.

| | **$S_1$** | **$S_2$** | **$S_3$** | **$S_4$** | **$M_1$** | **$M_2$** |
|---|---|---|---|---|---|---|
| **ΔE (eV)** | **1.65** | **1.83** | **1.86** | **2.03** | **2.06** | **1.75** |
| **f** | **0.86** | **1.27** | **0.05** | **0.27** | **2.15** | **2.03** |
| **Δμ (D)** | **9.80** | **13.44** | **13.58** | **17.24** | **4.72** | **8.38** |
| **Δp ($Å^3$)** | **1296** | **599** | **749** | **-973** | **864** | **690** |

**Supplementary Table 3.b:** Calculated energies for the lowest four singlet excited states ($S_1$ to $S_4$, denoted as ΔE) of dimer B of Y6, along with the corresponding oscillator strengths (f) for $S_0 \rightarrow S_n$ (n=1 to 4) transitions. The table also includes the variations in dipole moment (Δμ) and polarization (Δp) during these transitions. Additionally, similar values for the $S_1$ state are provided for the constituent monomeric units ($M_1$ and $M_2$), comprising dimer B of Y6.

| | **$S_1$** | **$S_2$** | **$S_3$** | **$S_4$** | **$M_1$** | **$M_2$** |
|---|---|---|---|---|---|---|
| **ΔE (eV)** | **1.63** | **1.63** | **1.69** | **1.75** | **1.75** | **1.75** |
| **f** | **0.02** | **0.39** | **0.00** | **3.62** | **2.03** | **2.03** |
| **Δμ (D)** | **10.59** | **10.37** | **0.15** | **0.11** | **8.41** | **8.41** |
| **Δp ($Å^3$)** | **59449** | **-58433** | **891** | **-552** | **687** | **687** |

**Supplementary Table 3.c:** Calculated energies for the lowest four singlet excited states ($S_1$ to $S_4$, denoted as ΔE) of dimer C of Y6, along with the corresponding oscillator strengths (f) for $S_0 \rightarrow S_n$ (n=1 to 4) transitions. The table also includes the variations in dipole moment (Δμ) and polarization (Δp) during these transitions. Additionally, similar values for the $S_1$ state are provided for the constituent monomeric units ($M_1$ and $M_2$), comprising dimer C of Y6.

| | $S_1$ | $S_2$ | $S_3$ | $S_4$ | $M_1$ | $M_2$ |
|---|---|---|---|---|---|---|
| **ΔE (eV)** | **1.60** | **1.70** | **1.78** | **1.90** | **1.75** | **1.81** |
| **f** | **0.137** | **1.033** | **1.922** | **0.201** | **2.10** | **1.82** |
| **Δμ (D)** | **30.41** | **17.05** | **10.86** | **26.08** | **12.21** | **7.42** |
| **Δp (Å$^3$)** | **722** | **203** | **1180** | **576** | **738** | **1255** |

**Supplementary Table 4.a:** Calculated energies for the lowest four singlet excited states ($S_1$ to $S_4$, denoted as ΔE) of dimer A of ITIC, along with the corresponding oscillator strengths (f) for $S_0 \rightarrow S_n$ (n=1 to 4) transitions. The table also includes the variations in dipole moment (Δμ) and polarization (Δp) during these transitions. Additionally, similar values for the $S_1$ state are provided for the constituent monomeric units ($M_1$ and $M_2$), comprising dimer A of ITIC.

| | $S_1$ | $S_2$ | $S_3$ | $S_4$ | $M_1$ | $M_2$ |
|---|---|---|---|---|---|---|
| **ΔE (eV)** | **1.85** | **1.86** | **2.10** | **2.10** | **1.86** | **1.86** |
| **f** | **5.66** | **0.01** | **0.00** | **0.00** | **2.80** | **2.81** |
| **Δμ (D)** | **0.27** | **0.05** | **100.91** | **100.55** | **0.07** | **0.17** |
| **Δp (Å$^3$)** | **1236** | **925** | **1765** | **1747** | **1063** | **1059** |

**Supplementary Table 4.b:** Calculated energies for the lowest four singlet excited states ($S_1$ to $S_4$, denoted as ΔE) of dimer B of ITIC, along with the corresponding oscillator strengths (f) for $S_0 \rightarrow S_n$ (n=1 to 4) transitions. The table also includes the variations in dipole moment (Δμ) and polarization (Δp) during these transitions. Additionally, similar values for the $S_1$ state are provided for the constituent monomeric units ($M_1$ and $M_2$), comprising dimer B of ITIC.

| | **$S_1$** | **$S_2$** | **$S_3$** | **$S_4$** | **$M_1$** | **$M_2$** |
|---|---|---|---|---|---|---|
| **ΔE (eV)** | **1.78** | **1.84** | **1.95** | **1.99** | **1.86** | **1.83** |
| **f** | **3.86** | **1.59** | **0.17** | **0.16** | **2.81** | **2.97** |
| **Δμ (D)** | **5.36** | **8.44** | **48.80** | **44.73** | **0.07** | **0.16** |
| **Δp (Å$^3$)** | **1745** | **2737** | **1414** | **-926** | **1059** | **869** |

- *<u>Part by part EA fitting for the different aggregate and non-interacting molecular regions based on Franck-Condon progressions.</u>*

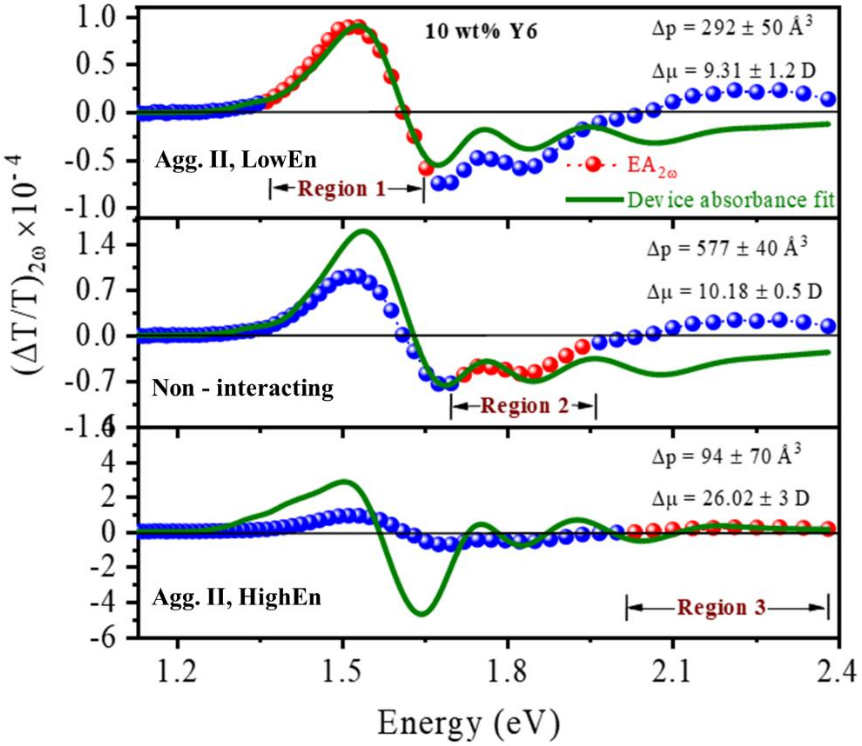


**Supplementary Fig. 9a: Electroabsorption spectra fitting of 10 wt% Y6**. Measured second harmonic (2ω) EA signals (transmission mode) of 10 wt% of Y6 in PVK fitted with Equation 4. The red symbols represent the fitted region of the EA signal, which corresponds to the low-energy aggregate (Agg II, LowEn), non-interacting, and high-energy aggregate (Agg II, HighEn), regions of thin film absorbance of 10 wt% Y6. The extracted Δμ (change in dipole moment) and Δp (change in polarizability) values of different aggregate configurations are shown as inset in the figure.

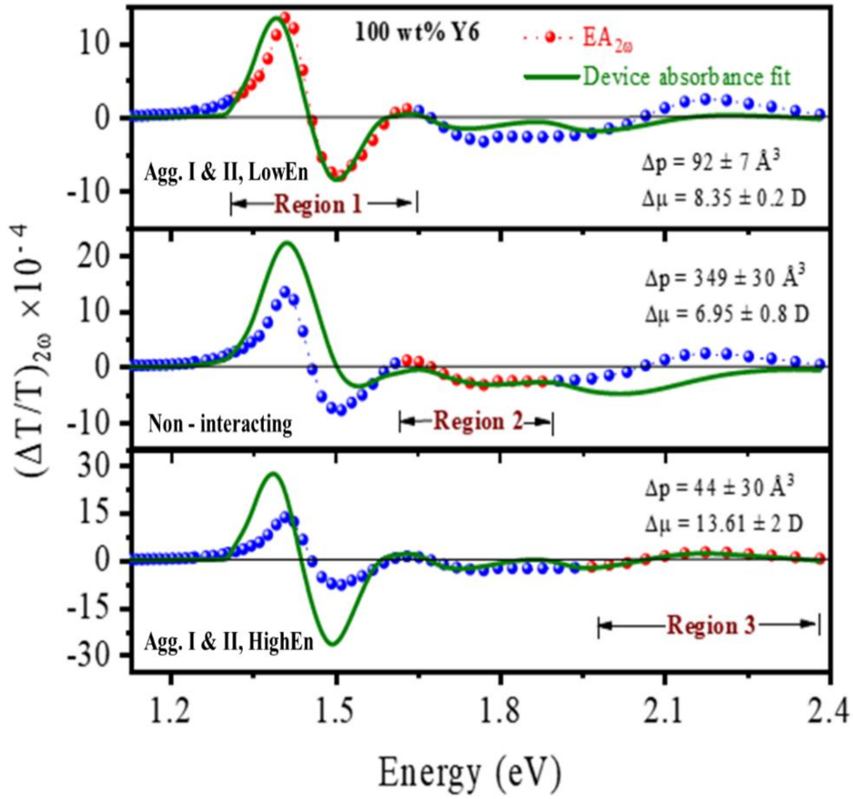


**Supplementary Fig. 9b: Electroabsorption spectra fitting of 100 wt% Y6**. Measured second harmonic (2ω) EA signals (transmission mode) of 100 wt% of Y6 fitted with Equation 4. The red symbols represent the fitted region of the EA signal, which corresponds to the low energy aggregate (Agg I & II, LowEn), non-interacting, and high energy aggregate (Agg I & II, HighEn), regions of thin film absorbance of pure Y6. The extracted Δμ (change in dipole moment) and Δp (change in polarizability) values of different samples are shown as inset in the figure.

## Supplementary Note 1: Stark Effect and the change in optical absorption

L. Sebastian et al. derived the electroabsorption fitting equation to correlate the Stark effect with the change of absorbance of a thin film under electrical field[9]. Here, we demonstrate such change in absorbance with respect to the measured first and second-harmonic transmission signals by electroabsorption spectroscopy. According to the Stark theory, each energy state will get shifted by ΔE under electric-field perturbation, which leads to changes in optical absorption[1]. The figure below shows the energy diagram with and without an electric field, where $E_0$ (F) and $E_0$ (0) represent the ground state energy with and without electric field, respectively. Also, $E_1$(F) and $E_1$(0) respectively represent the excited state energy with and without an electric field. $\Delta E_1$ and $\Delta E_0$ stand for the excited state and ground state energy shift. The Y-axis on the left is used to define the increasing direction of energy (eV).

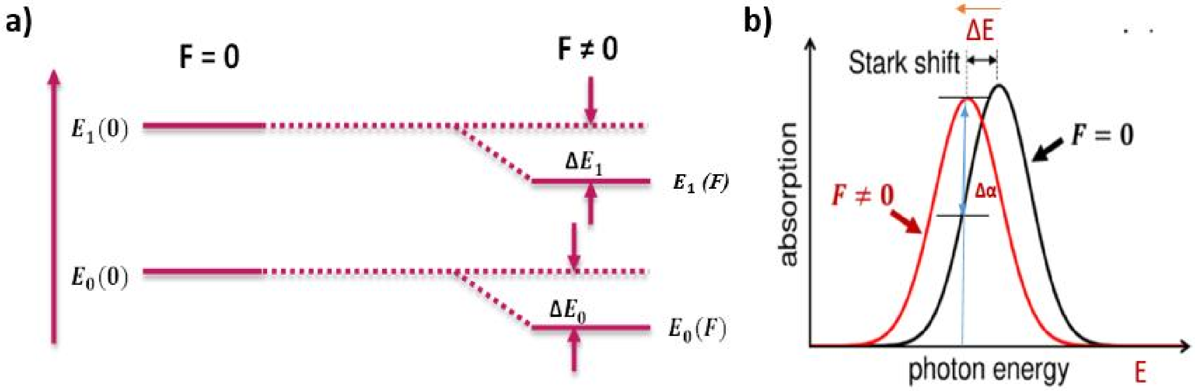


**Supplementary Fig. 10: a.** Energy diagram showing Stark effect **b.** Change in absorbance with and without the influence of external electric field.

Considering the overall change in transition energy ΔE from ground to excited state under an electrical field, i.e.,

ΔE = change in excitation energy with electric field, F.

= (excitation energy under electrical field) – (excitation energy without electrical field)

$= [E_1\,(F) - E_0\,(F)] - [E_1\,(0) - E_0\,(0)]$

$= [E_1\,(F) - E_1\,(0)] - [E_0\,(F) - E_0\,(0)]$

Therefore,

$$\Delta E = \Delta E_1 - \Delta E_0 \quad (1)$$

where

$\Delta E < 0$ corresponds to a red shift and $\Delta E > 0$ corresponds to a blue shift of the absorption spectrum, and the change in absorption coefficient $\Delta\alpha$ can be expressed as:

$$\Delta\alpha = \alpha_F(E) - \alpha(E)$$

$$= \alpha(E - \Delta E) - \alpha(E) \quad (2)$$

where $\alpha_F(E)$ and $\alpha(E)$ are the absorption coefficients with and without electric field.

According to the Stark effect, the energy level will shift under electric field F depending on its dipole moment and polarizability (material properties) in its equilibrium state.

$$E(F) \approx E(0) - \mu.F - \frac{1}{2} p.F^2$$

<u>Excited state</u> $$E_1(F) = E_1(0) - \mu_1 F - \frac{1}{2} p_1.F^2 \quad (3)$$

<u>Ground state</u> $$E_0(F) = E_0(0) - \mu_0 F - \frac{1}{2} p_0.F^2 \quad (4)$$

According to eqn. (1), $\Delta E = \Delta E_1 - \Delta E_0$

$$\Delta E = [E_1(F) - E_1(0)] - [E_0(F) - E_0(0)]$$

$$\Delta E = -[\mu_1 - \mu_0] F - \frac{1}{2} F^2 [p_1 - p_0] \quad (5)$$

$$\Delta E = -\Delta\mu F - \Delta p \frac{F^2}{2} \quad (6)$$

$\Delta\mu = (\mu_1 - \mu_0)$, difference in dipole moment (unit- Debye) between ground and excited energy states.

$\Delta p = (p_1 - p_0)$, difference in polarizability (unit – $cm^3$) between ground and excited energy states.

The change of absorption coefficient Δα of a thin film can be expressed as a Taylor series:

$$\Delta\alpha \approx -\frac{\partial\propto}{\partial E}(\Delta E) + \frac{1}{2}\frac{\partial^2\propto}{\partial E^2}(\Delta E)^2 - \frac{1}{6}\frac{\partial^3\propto}{\partial E^3}(\Delta E)^3 + \ldots \quad (7)$$

$\frac{\partial\propto}{\partial E}$ , $\frac{\partial^2\propto}{\partial E^2}$ and $\frac{\partial^3\propto}{\partial E^3}$ are the first, second, and third derivatives of the absorption coefficient, respectively. To derive the electroabsorption equation in terms of Δμ and Δp, we can substitute eqn. (5) into eqn. (6),

$$\Delta\alpha = -\frac{\partial\propto}{\partial E}(-\Delta\mu F - \Delta p\frac{F^2}{2}) + \frac{1}{2}\frac{\partial^2\propto}{\partial E^2}(-\Delta\mu F - \Delta p\frac{F^2}{2})^2 - \frac{1}{6}\frac{\partial^3\propto}{\partial E^3}(-\Delta\mu F - \Delta p\frac{F^2}{2})^3 \quad (8)$$

In eqn. (7), as we focus on the first and second derivative terms, the higher-order derivatives are neglected, i.e.:

$$\Delta\alpha = -\frac{\partial\propto}{\partial E}(-\Delta\mu F - \Delta p\frac{F^2}{2}) + \frac{1}{6}\frac{\partial^2\propto}{\partial E^2}(\Delta\mu^2 F^2) - [\Delta p\ \frac{F^2}{2}]^2) \quad (9)$$

In order to formulate the first and second harmonic EA equations, eqn. (8) is substituted by the applied electric field with the superposition of an AC and DC components as $F = F_{dc} + F_{ac}$ sin ωt.

$$\Delta\alpha = -\frac{\partial\propto}{\partial E}(((-\Delta\mu(F_{dc} + F_{ac}\ \sin\ \omega t) - \frac{\Delta p}{2}[{F_{dc}}^2 + 2F_{dc}F_{ac}\ \sin\ \omega t + {F_{ac}}^2 sin^2\ \omega t) + \frac{1}{6}\frac{\partial^2\propto}{\partial E^2}(\Delta\mu^2[{F_{dc}}^2 + 2F_{dc}F_{ac}\ \sin\omega t + {F_{ac}}^2 sin^2\ \omega t]) \quad (10)$$

In eqn. (9), the DC, sin ωt, and $sin^2$ ωt terms contribute to the zero, first, and second harmonic signals, respectively, i.e.:

- Zeroth harmonic terms

$$\Delta\alpha_{0\omega} = \frac{\partial\propto}{\partial E}(\Delta\mu\,(F_{dc} + \frac{\Delta p}{2}[{F_{dc}}^2 + \frac{{F_{ac}}^2}{2}])) + \frac{1}{6}\frac{\partial^2\propto}{\partial E^2}(\Delta\mu^2[{F_{dc}}^2 + \frac{{F_{ac}}^2}{2}]) \quad (11)$$

- First harmonic terms

$$\Delta\alpha_{1\omega} = \frac{\partial\propto}{\partial E}((\Delta\mu(F_{ac}\ \sin\omega t) + \frac{\Delta p}{2}(2F_{dc}F_{ac}\ \sin\omega t)) + \frac{1}{3}\frac{\partial^2\propto}{\partial E^2}(\Delta\mu^2(F_{dc}F_{ac}\ \sin\omega t) \quad (12)$$

- Second harmonic terms

$$\varDelta\alpha_{2\omega} = -\ \frac{\partial\propto}{\partial E}\ (\ \frac{\Delta \mathrm{p}}{2} \times \frac{{F_{ac}}^2}{2}\ \sin\,[2\omega t\ +\ \frac{\pi}{2}]\ -\ \frac{1}{6}\ \frac{\partial^2\propto}{\partial E^2}\ (\Delta\mu^2(\frac{{F_{ac}}^2}{2}\ \sin\,[2\omega t\ +\ \frac{\pi}{2}])$$

(13)

By rearranging the terms related to the first and second harmonic EA equations,

$$\varDelta\alpha_{1\omega} = \frac{\partial\propto}{\partial E}\ (\Delta \mathrm{p})\ F_{dc}F_{ac}\ \sin\omega t + \frac{1}{3}\frac{\partial^2\propto}{\partial E^2}(\Delta\mu^2)F_{dc}F_{ac}\ \sin\omega t + \frac{\partial\propto}{\partial E}(\Delta\mu(F_{ac}\sin\omega t)) \qquad (14)$$

$$\varDelta\alpha_{2\omega} = -\ \frac{1}{4}\frac{\partial\propto}{\partial E}\ (\Delta \mathrm{p}){F_{ac}}^2\sin\,[2\omega t\ +\ \frac{\pi}{2}\ ] - \frac{1}{12}\ \frac{\partial^2\propto}{\partial E^2}\ (\Delta\mu^2){F_{ac}}^2\sin\,[2\omega t\ +\ \frac{\pi}{2}] \qquad (15)$$

The last term $\frac{\partial\propto}{\partial E}(\Delta\mu\ (F_{ac}\ \sin\omega t))$ in the first harmonic EA equation (eqn.13) is usually omitted in isotropic media where dipoles are randomly oriented. Δμ is a vector which will cancel out upon averaging the ensembles. In the manuscript, we use Δμ' to represent a preferred orientation of dipoles to avoid confusion with $\Delta\mu^2$.

As EA is measured in transmission mode (T), where T = A $e^{-\alpha d}$

$$\Delta T = A\ e^{-\alpha d}.(-\varDelta\alpha d)$$

$$\Delta T = T\ .(-\varDelta\alpha d)$$

$$\frac{\Delta T}{T} = -\Delta\alpha.\ d \qquad (16)$$

where d is the thickness of the active layer.

Substituting eqn. (16) into eqn. (14) and (15), i.e.:

$$\frac{\Delta T}{T}_{1\omega} = -\ [\ \frac{\partial\alpha}{\partial E}(\Delta \mathrm{p}) + \frac{1}{3}\ \frac{\partial^2\alpha}{\partial E^2}(\Delta\mu^2)]\frac{V_{dc}V_{ac}}{d}\ \sin\,[\omega t] \qquad (17)$$

$$\frac{\Delta T}{T}_{2\omega} = [\ \frac{1}{4}\frac{\partial\alpha}{\partial E}\ (\Delta \mathrm{p}) + \frac{1}{12}\ \frac{\partial^2\alpha}{\partial E^2}\ (\Delta\mu^2)]\ \frac{{V_{ac}}^2}{d}\ \sin\,[2\omega t + \frac{\pi}{2}] \qquad (18)$$

Equations (17) and (18) can be further expressed in terms of device absorbance $A_D$ by replacing the absorption coefficient α as described below:

Converting absorption coefficient into device absorbance ($A_D$),

$$A_D = \log \frac{T_0}{T} = log_{10}\left(\frac{T_0}{T_0.e^{\alpha.d}}\right)$$

$$= log_{10}\left(e^{\alpha.d}\right) \;\; = \alpha.\text{d}.\; log_{10}\;(\text{e}),$$

$$\approx 0.43.\alpha.\text{d},\;\; log_{10}\;(\text{e}) = 0.43$$

$$\alpha = \frac{A_D}{0.43\times d} \tag{19}$$

Rewriting the first and second harmonic EA equations in terms of transmittance (T) and $A_D$:

- <u>First harmonic EA fitting equation</u>

$$\frac{\Delta T}{T}_{1\omega} = -\;\left[\frac{\partial A_D}{\partial E}\;(\Delta \text{p})\; +\; \frac{1}{3}\;\frac{\partial^2 A_D}{\partial E^2}\;(\Delta \mu^2)\right]\frac{V_{dc}V_{ac}}{0.43d^2}\;\sin\,[\omega t] \tag{20}$$

- <u>Second harmonic EA fitting equation</u>

$$\frac{\Delta T}{T}_{2\omega} = \left[\frac{1}{4}\frac{\partial A_D}{\partial E}\;(\Delta \text{p}) + \frac{1}{12}\;\frac{\partial^2 A_D}{\partial E^2}\;(\Delta \mu^2)\right]\frac{{V_{ac}}^2}{0.43d^2}\;\sin\,[2\omega t\; +\; \frac{\pi}{2}] \tag{21}$$

In addition, in case there is no preferred dipole orientation, the first and second harmonic should have the same spectral characteristics and their ratio is simply proportional to the applied DC and AC voltage bias.

Eqn. (20) divided by eqn. (19) implies:

$$\frac{\frac{\Delta T}{T}_{2\omega}}{\frac{\Delta T}{T}_{1\omega}} = \frac{V_{ac}}{-4\,V_{dc}} \tag{22}$$

**Supplementary Note 2: Dipole orientation in thin films.**

Supplementary Fig. 11a shows the DC bias-dependent EA spectra of 10 wt% and pure Y6 and ITIC devices measured at the first harmonic $1\omega$ of the AC modulation electrical field. The first and second harmonic DC bias dependence results of devices with other loading ratios are summarized in Supplementary Fig. 11 (b) and (c). In the case of Y6, $\left(\frac{\Delta T}{T}\right)_{1\omega}$ is proportional to $V_{dc}$ in the 10 wt% Y6 devices, but the spectrum changes drastically in pure Y6 device with non-zero crossing points at 1.45 eV and 1.63 eV. In the case of ITIC, the $EA_{1\omega}$ spectra of both devices with low and high loading ratios are directly proportional to $V_{dc}$ with the same spectral line shape as in the $EA_{2\omega}$ spectra. Equation 23 below shows the change in device transmission derived from the Stark effect at the first harmonic $1\omega$ of the AC modulation electrical field. Details of the derivation are described in Supplementary Note 1.

$$\frac{\Delta T}{T}_{1\omega} =- \left[(\Delta p)\,\frac{\partial A_D}{\partial E} + \frac{1}{3}\,(\Delta\mu^2)\frac{\partial^2 A_D}{\partial E^2}\right]\frac{V_{ac}V_{dc}}{0.43d^2}\sin(\omega t) - \frac{1}{0.43}\,(\Delta\mu')\frac{\partial A_D}{\partial E}\,\left(\frac{V_{ac}}{d}\sin(\omega t)\right) \quad (23)$$

where $\Delta\mu'$ is the linear average of the dipole moment between the ground and excited states; therefore, the last term on the right-hand side (RHS) is usually omitted in the case of randomly oriented molecules in an isotropic media. Then, $\left(\frac{\Delta T}{T}\right)_{1\omega}$ will be proportional to the DC potential $V_{dc}$ across the organic film, and this equation has been widely used to determine the built-in potential in organic thin -ilm devices[10–12]. Moreover, without the last term on the RHS, the spectral line shape of the measured first and second harmonic spectra should be essentially identical, only scaled by a ratio as a function of $V_{dc}$ and the ac modulating voltage $V_{ac}$, i.e. $\left(\frac{\Delta T}{T}\right)_{2\omega}/\left(\frac{\Delta T}{T}\right)_{1\omega} =-\frac{V_{ac}}{4V_{dc}}$. On the other hand, as shown in the calculation results in Supplementary Fig. 11d, if there is a preferred dipole orientation with non-zero $\Delta\mu'$, the last term on the RHS would have a large influence on the overall $EA_{1\omega}$ spectral line shape. In our previous study, we had also shown that for an electrically poled molecular thin film with the molecules mostly aligned in one direction, the $EA_{1\omega}$ spectrum was only dependent on the last term on the RHS and

became dc bias independent[13]. By taking the non-zero crossing point at 1.63eV in the pure Y6 device, we obtain the $\Delta\mu'$ value around 0.04 D. This small value might not directly reflect the dipole moment of the molecules with a preferred orientation, as both the populations of the randomly aligned and preferentially aligned molecules as well as the direction between the dipole moment and the electrical field are unknown. However, the increasing non-zero crossing spectral characteristics while increasing the loading ratio in Y6 devices strongly support that there is a preferred molecular orientation in the Y6 thin film. It is consistent with the GIWAXS results at different loadings of Y6, as shown in Supplementary Fig. 5 where a clear face-on orientation is revealed in the 2D patterns, and the results of recent MD simulations on a Y6 thin film[14].

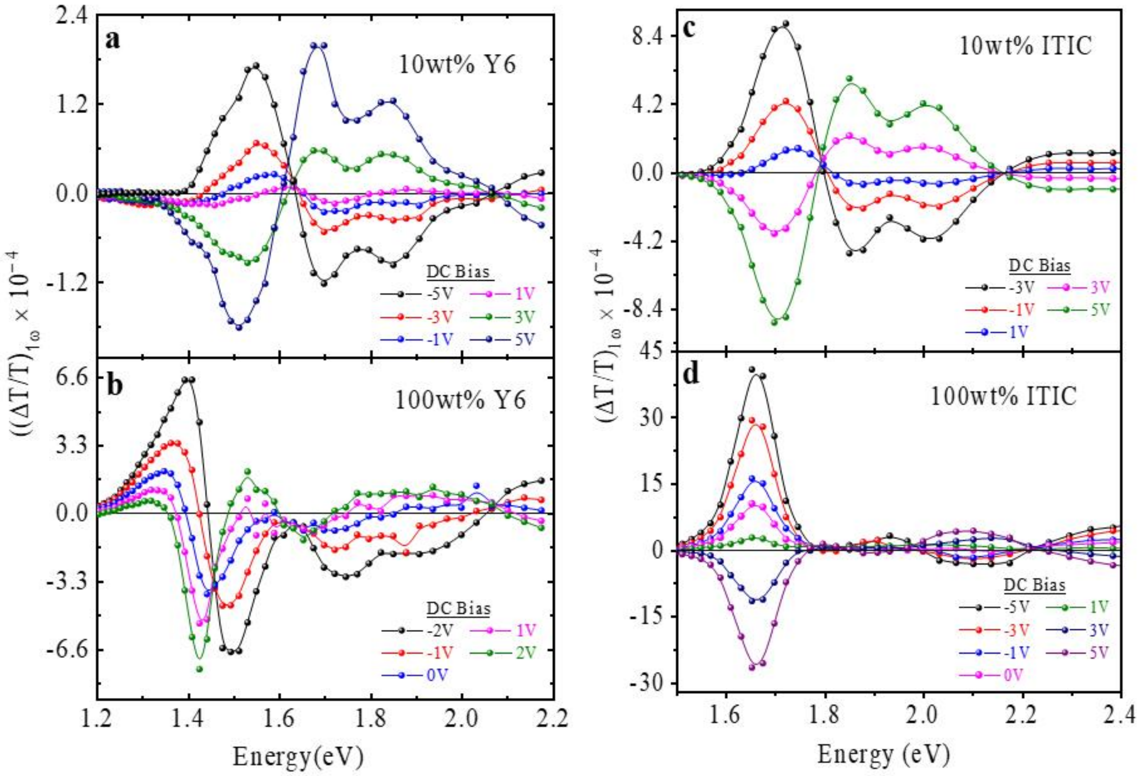


**Supplementary Fig. 11a: First harmonic DC bias dependence EA Spectra of dispersed and pristine Y6 and ITIC thin films.** The measured first harmonic EA signals at different loading ratios (10 wt% and 100 wt%) of **a, b** Y6 and **c, d** ITIC in a PVK matrix at different applied DC bias. The DC bias dependence of the first harmonic EA signals in 100 wt% Y6 shows asymmetric spectral characteristics.

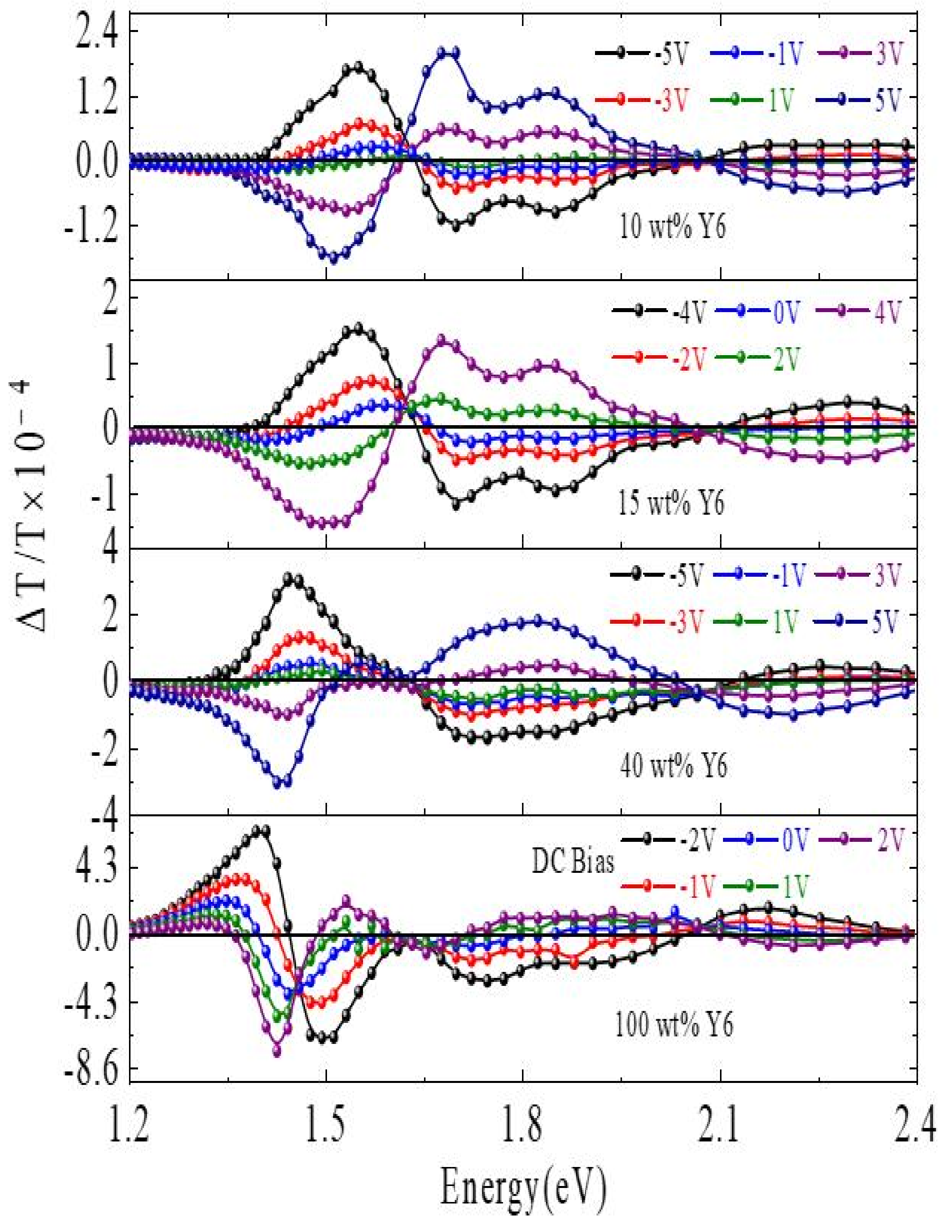


**Supplementary Fig. 11b:** First harmonic DC bias dependence of EA signals at different loading ratios of Y6 in a PVK matrix.

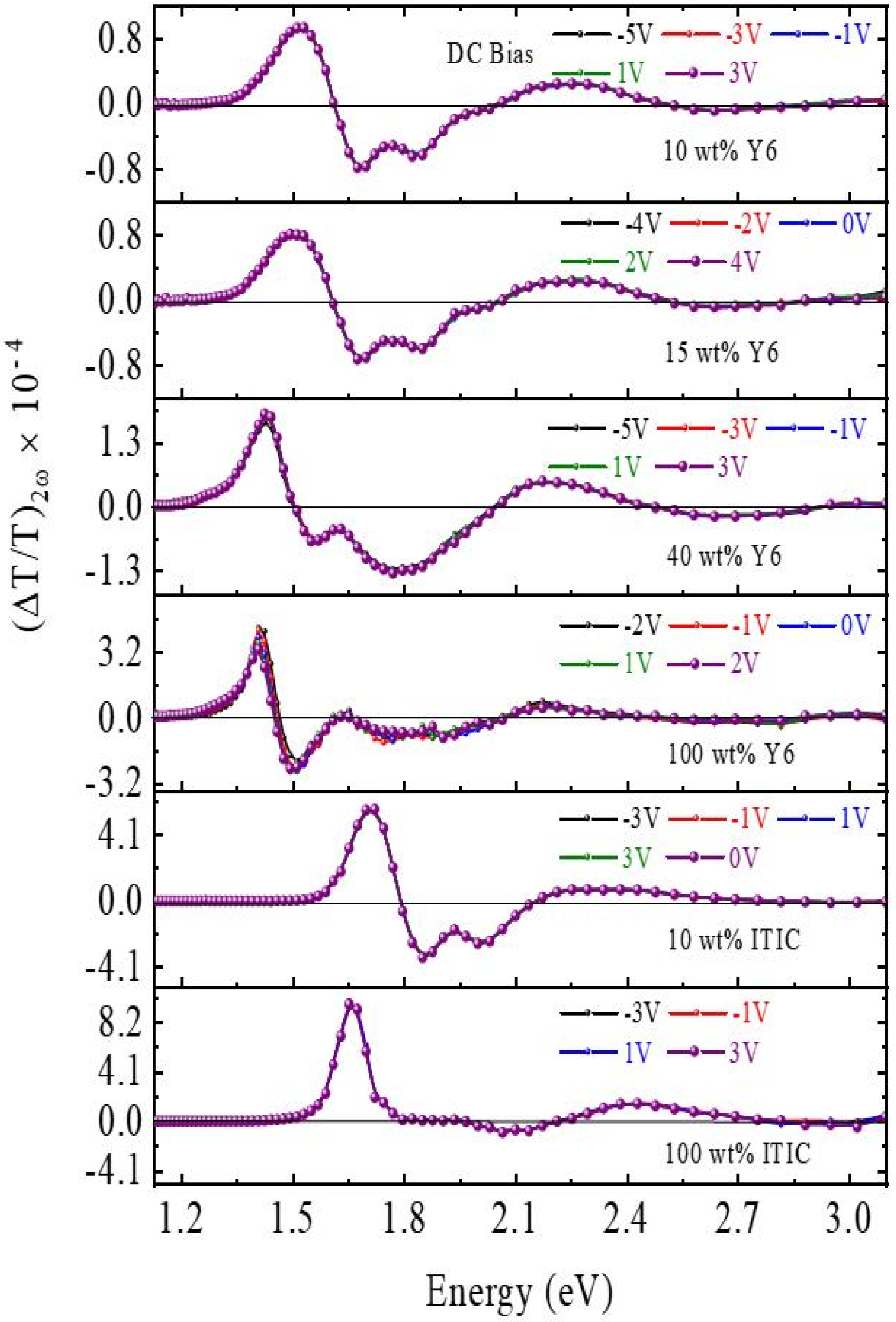


**Supplementary Fig. 11c:** Second harmonic DC bias dependence of EA signals at different loading ratios of Y6 and ITIC in PVK polymer matrix. Second harmonic EA signals are DC bias independent as shown in Equation 4 in the manuscript. Also, the second harmonic EA signals will be insensitive to any static electric field[15].

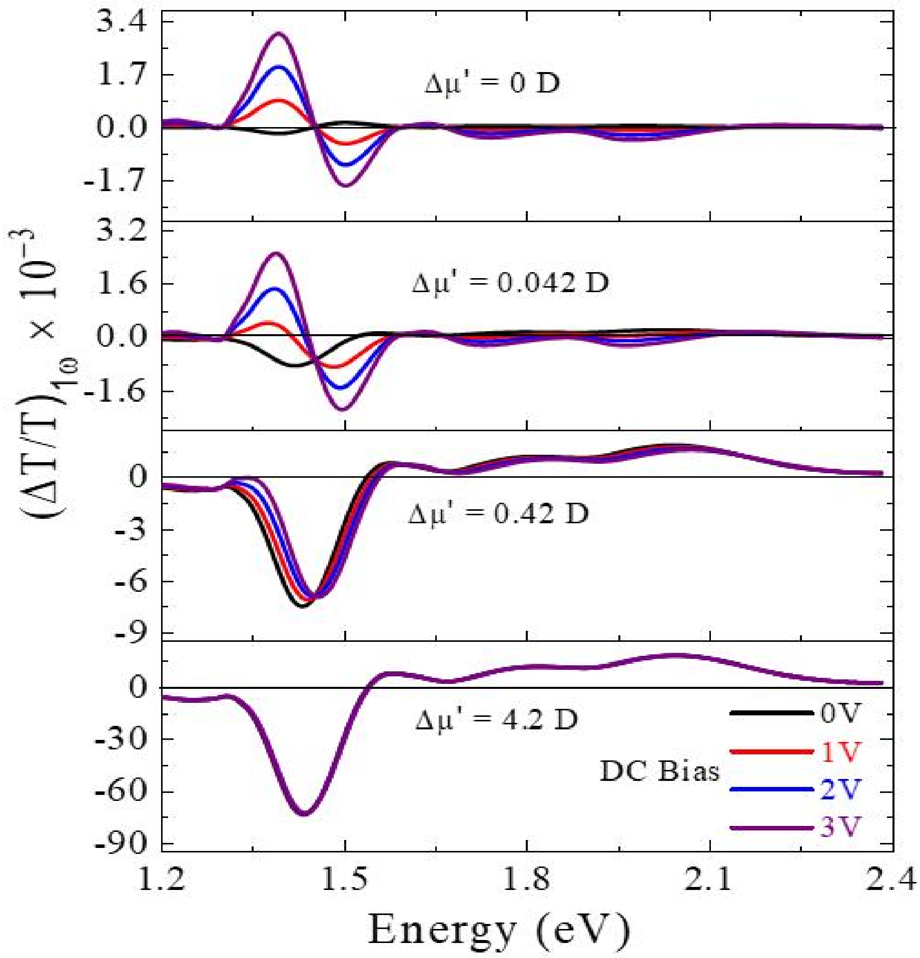


**Supplementary Fig. 11d:** Calculated first harmonic DC bias dependence of EA spectra of pristine Y6 with different values of anisotropic or static dipole moment. We can observe the change in the isosbestic point as the value of the static dipole moment changes from 0 to 4.2 Debye.

At 0 Debye, the isosbestic point is on the x-axis and represents randomly oriented dipoles in thin films. This can be observed in a low-concentration sample of Y6 in PVK. In pristine Y6 film, the static dipole moment is around 0.042 D, and the spectral lineshape changes, which indicates a directional packing of molecules in pristine Y6[10-12]. As we further increase the static dipole moment (D), the first harmonic signals become more DC bias independent, which shows fully directionally oriented molecules with strong $(EA)_{1\omega}$ signal intensity in the order of $10^{-2}$ as reported in literature[13].

**Supplementary Table 5:** Calculation of linear dipoles from first harmonic DC bias dependence of EA signals at different loading ratios of Y6 in a PVK matrix. (Here, Δp is expressed in $cm^3$ units with 1 $cm^3$ equivalent to 1×$10^{24}$ A³).

| wt% of Y6 | Non-zero crossing point (eV) | Δp × $10^{-22}$ ($cm^3$) | Δμ (D) | Δμ/Δp × $10^{22}$ ($D.cm^{-3}$) | $\frac{\partial A_D}{\partial E}$ | $\frac{\partial^2 A_D}{\partial E^2}$ | $\frac{\partial A_D}{\partial E}$ (Δp) × $10^{-19}$ | $\frac{1}{3}\frac{\partial^2 A_D}{\partial E^2}$ (Δμ²) × $10^{-19}$ | $\frac{\Delta T}{T}$× $10^{-4}$ | Δμ' (D) |
|---|---|---|---|---|---|---|---|---|---|---|
| 10 wt% | 1.63 | 3.07 | 9.74 | 3.17 | 0.58 | -18.5 | 1.24 | 2.52 | 0.147 | -0.02 |
| 15 wt% | 1.63 | 2.13 | 9.41 | 4.42 | 0.66 | -22.17 | 0.98 | -2.81 | 0.29 | -0.04 |
| 40 wt% | 1.63 | 1.55 | 8.21 | 5.26 | -0.08 | -5.6 | -0.09 | -0.54 | -0.17 | -0.19 |
| 100 wt% | 1.46 | 0.95 | 8.46 | 8.91 | 8.58 | -62.91 | 5.61 | -6.45 | -3.35 | 0.04 |

**Supplementary Note 3: Fitting procedure of $EA_{2\omega}$ spectrum using Gaussian deconvolution.**

The thin-film absorbance data were measured from UV-vis absorption spectroscopy and then deconvoluted as Gaussian bands using the Origin software. Then, we estimate the peak position and excitation energy of the first Gaussian band, which corresponds to the $S_1$ state. Also, we calculate the first and second derivatives of device absorbance $(A_D)$ to fit the $EA_{2\omega}$ data using equation (20). For the lowest loading ratio of Y6 in PVK i.e., 1wt%, we used thin film absorbance derivatives to do the fitting analysis.

$$\frac{\Delta T}{T}_{2\omega} = [\frac{1}{4}\frac{\partial A_D}{\partial E}(\Delta p) + \frac{1}{12}\frac{\partial^2 A_D}{\partial E^2}(\Delta \mu^2)]\frac{{V_{ac}}^2}{0.43d^2}\sin[2\omega t + \frac{\pi}{2}] \quad (21)$$

This second harmonic EA fitting procedure is used to calculate Δp and Δμ values to verify the excitonic nature of the $S_1$ state, either Frenkel or CT type.

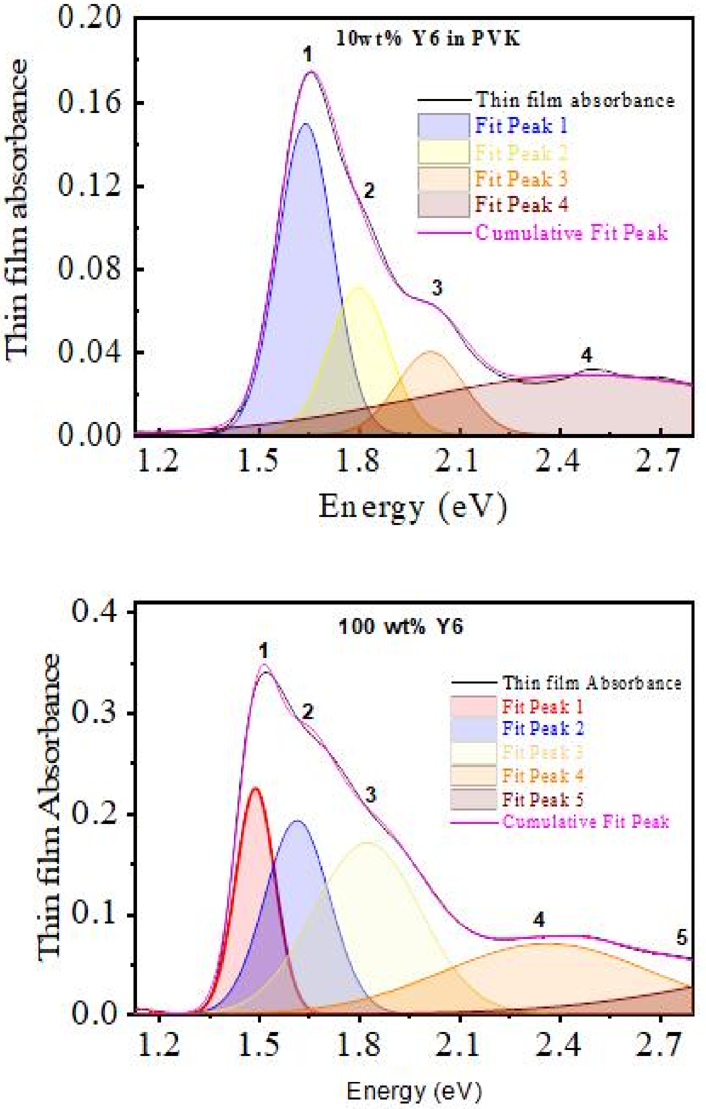


**Supplementary Fig. 12:** Thin-film absorbance fitting using Gaussian deconvolution.

**Supplementary Note 4: Four-State Model.** To model the variation in dipole moment and polarization as a function of $\Delta E_{LE\text{-}CT}$, we considered the following four-state model[16,17].

$$H = \begin{pmatrix} E_{M_1}^{LE} & d & t_1 & t_2 \\ d & E_{M_2}^{LE} & t_2 & t_1 \\ t_1 & t_2 & E_{CT}^{M_1^+M_2^-} & 0 \\ t_2 & t_1 & 0 & E_{CT}^{M_1^-M_2^+} \end{pmatrix}$$

In this matrix, $E_{M_1}^{LE}$ and $E_{M_2}^{LE}$ are the LE (Frenkel) excitation energies for monomer 1 ($M_1$) and monomer 2 ($M_2$) within the dimer, respectively; $E_{CT}^{M_1^+M_2^-}$ and $E_{CT}^{M_1^-M_2^+}$ represent the charge transfer energies for excitation from $M_1$ to $M_2$ or vice versa. The effect of the electric field (F) is described as follows:

$$E_{M_1}^{LE} = E_{M_1}^{LE}(0) - \Delta\mu_{M_1}^{LE} \times F - 0.5 \times \Delta p_{M_1}^{LE} \times F^2 \quad (24)$$

$$E_{M_2}^{FE} = E_{M_2}^{LE}(0) - \Delta\mu_{M_2}^{LE} \times F - 0.5 \times \Delta p_{M_2}^{LE} \times F^2 \quad (25)$$

$$E_{CT}^{M_1^+M_2^-} = E_{CT}^{M_1^+M_2^-}(0) - \Delta\mu_{CT}^{M_1^+M_2^-} \times F - 0.5 \times \Delta p_{CT}^{M_1^+M_2^-} \times F^2 \quad (26)$$

$$E_{CT}^{M_1^-M_2^+} = E_{CT}^{M_1^-M_2^+}(0) - \Delta\mu_{CT}^{M_1^-M_2^+} \times F - 0.5 \times \Delta p_{CT}^{M_1^-M_2^+} \times F^2 \quad (27)$$

Based on previous DFT calculations[7,18], we used the following model parameters: $\Delta\mu_{M_1}^{LE} = \Delta\mu_{M_2}^{LE}$= 8 D; $\Delta\mu_{CT}^{M_1^-M_2^+} = \Delta\mu_{CT}^{M_1^+M_2^-}$ =10 D; $\Delta\mu_{CT}^{M_1^-M_2^+} = \Delta p_{CT}^{M_1^-M_2^+} = 0$; $\Delta p_{M_1}^{LE} = \Delta p_{M_2}^{LE} = 950$ Å$^3$ ; $E_{CT}^{M_1^+M_2^-}(0) - E_{CT}^{M_1^-M_2^+}(0) = 50\ meV;\ d = 50\ meV;$ and $t_1$ =$t_2$ =100 meV. The energies of the eigenstates of the four-state model were calculated for $\Delta E_{CT-LE}$ values in the range from 0.3 eV to -0.3 eV as a function of electric field. The Δμ and Δp values were then derived by computing the first and second derivatives of the $S_1$ energy as a function of electric field.

## Supplementary References


1. Stark, J. Observation of the Separation of Spectral Lines by an Electric Field. Nature **92**, 401 (1913).

2. Aldrich, T.J., Matta, M., Zhu, W., Swick, S.M., Stern, C.L., Schatz, G.C., Facchetti, A., Melkonyan, F.S., Marks, T.J., Fluorination Effects on Indacenodithienothiophene Acceptor Packing and Electronic Structure, End-Group Redistribution, and Solar Cell Photovoltaic Response. *J. Am. Chem. Soc.* **141** (7), 3274-3287 (2019).

3. Kasha, M. Characterization of electronic transitions in complex molecules. *Discuss. Faraday Soc.* **9**, 14–19 (1950).

4. Brédas, J. L., Beljonne, D., Coropceanu, V. & Cornil, J. Charge-transfer and energy-transfer processes in π-conjugated oligomers and polymers: A molecular picture. *Chem. Rev.* **104**, 4971–5003 (2004).

5. Gao, J. *et al.* Over 16.7 % efficiency of ternary organic photovoltaics by employing extra PC71BM as morphology regulator. *Sci. China Chem.* **63**, 83–91 (2020).

6. Kroh, D. *et al.* Identifying the Signatures of Intermolecular Interactions in Blends of PM6 with Y6 and N4 Using Absorption Spectroscopy. *Adv. Funct. Mater.* **32**, 2205711 (2022).

7. Zhang, G. *et al.* Delocalization of exciton and electron wavefunction in non-fullerene acceptor molecules enables efficient organic solar cells. *Nat. Commun.* **11**, 3943 (2020).

8. Zhu, W. *et al.* Crystallography, Morphology, Electronic Structure, and Transport in Non-Fullerene/Non-Indacenodithienothiophene Polymer:Y6 Solar Cells. *J. Am. Chem. Soc.* **142**, 14532–14547 (2020).

9. Sebastian, L., Weiser, G. & Bassler, H. Charge transfer transitions in solid tetracene and pentacene studied by electroabsorption. Chem. Phys. **61**, 125–135 (1981).

10. Siebert-Henze, E. *et al.* Electroabsorption studies of organic p-i-n solar cells: Increase of the built-in voltage by higher doping concentration in the hole transport layer. *Org. Electron.* **15**, 563–568 (2014).

11. Siebert-Henze, E. *et al.* Built-in voltage of organic bulk heterojunction p-i-n solar cells measured by electroabsorption spectroscopy. *AIP Adv.* **4**, 0–10 (2014).

12. Stampor, W. Internal electric fields in vacuum-evaporated organic films as studied by electroabsorption spectroscopy. *Chem. Phys.* **334**, 216–223 (2007).

13. Liu, T. *et al.* Record-high near-band-edge optical nonlinearities and two-level model

correction of poled polymers by spectroscopic Electromodulation and ellipsometry. *Sci. China Chem.* **65**, 584–593 (2022).

14. Kupgan, G., Chen, X.K. & Brédas, J.L. Molecular packing of non-fullerene acceptors for organic solar cells: Distinctive local morphology in Y6 vs. ITIC derivatives. *Mater. Today Adv*. **11**, 100154 (2021).

15. Lane, P. A. *et al.* Electroabsorption studies of phthalocyanine/perylene solar cells. *Sol. Energy Mater. Sol. Cells* **63**, 3–13 (2000).

16. Petelenz, P., Mixing of Frenkel excitons with charge transfer states in the neighbourhood of a charged defect, *Chemical Physics Letters*, **47** (3), 603-605 (1977).

17. Petelenz, P., Theoretical models for electro-absorption spectroscopy, *Organic Electronics*, **5**, (1–3), 115-127 (2004).

18. Price, M. B. *et al.* Free charge photogeneration in a single component high photovoltaic efficiency organic semiconductor. *Nat. Commun.* **13**, 2827 (2022).
